# Mining Microbial Dark Matter: Advanced Cultivation Techniques for Bioactive Compound Discovery

**DOI:** 10.3390/ph18101583

**Published:** 2025-10-20

**Authors:** Minhui Ji, Bingda Ma, Jiayu Dong, Shan Liu, Ying Shi, Meiting Bu, Luoyi Wang, Ling Liu

**Affiliations:** 1 State Key Laboratory of Microbial Diversity and Innovative Utilization, Institute of Microbiology, Chinese Academy of Sciences, Beijing 100101, China; jiminhui23@mails.ucas.ac.cn (M.J.); mabingda23@mails.ucas.ac.cn (B.M.); dongjiayu23@mails.ucas.ac.cn (J.D.); shiying22@mails.ucas.ac.cn (Y.S.); bumeiting24@mails.ucas.ac.cn (M.B.); 2 College of Life Sciences, University of Chinese Academy of Sciences, Beijing 100049, China; 3 Beijing Key Laboratory of Genetic Element Biosourcing & Intelligent Design for Biomanufacturing, Institute of Microbiology, Chinese Academy of Sciences, Beijing 100101, China

**Keywords:** natural products, uncultured microorganisms, cultivation strategies, metagenomics, single-cell genomics, synthetic biology

## Abstract

The vast majority of microorganisms in the environment remain uncultured using conventional laboratory techniques, representing an immense untapped reservoir of genetic and chemical diversity. Recent innovations in cultivation strategies, combined with advances in metagenomics, single-cell genomics, and synthetic biology, have opened new avenues for accessing and harnessing bioactive natural products from these previously inaccessible microorganisms. This review highlights recent methodological and technological advancements in the cultivation and identification of novel microorganisms, and showcases the resulting discoveries of new natural products, demonstrating their potential for drug development.

## 1. Introduction

The exploration of natural products from microorganisms has been a major driver of pharmaceutical and biotechnological innovation. With the escalating threat of global antimicrobial resistance, there is an urgent need for new therapeutics with novel mechanisms of action to combat drug-resistant strains effectively [1]. Historically, the discovery of microbial natural products has predominantly relied on the cultivation of microorganisms in controlled laboratory environments [2]. However, traditional cultivation-based approaches have only scratched the surface of microbial diversity, leaving the vast majority of microorganisms—and their untapped chemical and biological potential—largely unexplored [3]. This highlights the necessity for novel strategies to delve deeper into the microbial world and unlock its full therapeutic potential [4].

Uncultured microorganisms, particularly those inhabiting unique and extreme environments, are believed to harbor novel biosynthetic pathways capable of producing structurally diverse and biologically active secondary metabolites, which are crucial for the development of antibiotics, anticancer agents, and other therapeutic compounds [5]. However, the challenge of culturing the majority of these microorganisms has hindered the discovery of new natural products. To overcome this hurdle, innovative cultivation strategies, including co-cultivation [6], diffusion chambers [7], and microfluidic cultivation [8] have been developed to enable the growth of previously uncultured microorganisms. Additionally, metagenomics has emerged as a powerful tool, enabling the direct extraction and analysis of genetic material from environmental samples, leading to the identification of new biosynthetic gene clusters [9]. Furthermore, single-cell genomics has advanced our understanding by providing detailed insights into the metabolic capabilities of individual microorganisms [10]. Meanwhile, synthetic biology has played a pivotal role in reconstructing and expressing complex biosynthetic pathways in heterologous hosts [11]. The integration of these advanced technologies with high-throughput screening and analytical techniques has significantly accelerated the discovery of novel natural products with promising therapeutic applications.

In this review, we aim to summarize the innovative strategies that have been employed to uncover uncultivated microorganisms from diverse environmental niches. We also highlight recent breakthroughs in the discovery of natural products and underscore the immense potential of these uncultured microbial resources. Consequently, employing the key words “uncultivated microorganisms” + “isolation and cultivation methods”, “microbial dark matter” + “cultivation”, and “uncultivated/difficult-to-cultivate microorganisms” + “natural products”, this review provides insights into the literature published from 2010 to 2024 by searching on PubMed and Web of Science databases. By unlocking the chemical diversity harbored by these elusive microorganisms, we can address critical challenges in medicine and biotechnology, paving the way for the development of new drugs, agrochemicals, and industrial biocatalysts.

## 2. Cultivation Strategies for Uncultured Microorganisms

The cultivation of microorganisms from diverse environments has been a slow process, hindered by a multitude of intricate factors. The natural habitats that support microbial life are challenging to replicate in laboratory conditions due to varying parameters such as pH, temperature, and pressure [12]. Additionally, the specific nutritional demands and growth factors of many microbes remain poorly understood. Dormant states in microbial life cycles and the essential role of microbial interactions, including both interspecies and intraspecific relationships, add significant layers of complexity to cultivation efforts [13]. While interspecies interactions such as symbiosis, competition, and cross-feeding are well-recognized for their influence on microbial growth and activity, intraspecific interactions—including quorum sensing, cooperation, and genetic exchange within the same species—also profoundly affect physiological states and cultivation outcomes. Moreover, environmental factors such as nutrient gradients, oxygen availability, and spatial structure can modulate these interactions, either by promoting cooperation or exacerbating competition. To address these challenges, innovative cultivation technologies are being developed that aim to mimic ecological conditions and microbial social dynamics, thereby helping to unlock the full potential of the microbial world.

### 2.1. Classical Cultivation Strategies and Methods

Classical microbiological methods continue to play a significant role in the cultivation of microorganisms, providing a reliable foundation for isolating various targeted species [4]. These methods primarily rely on the physiological, phenotypic, and functional characteristics of microorganisms to distinguish between different species. To enrich specific microbial taxa, a variety of strategies have been employed, including the incorporation of specific nutritional factors such as zincmethylphyrins, coproporphyrins, short-chain fatty acids, and iron oxides that fulfill the unique metabolic requirements of fastidious uncultured microbes [14,15,16,17,18], crafting nutrient media with selective properties [19,20,21,22,23], manipulating physicochemical conditions to favor certain species [24,25,26,27], and utilizing bio-devices such as biofilm reactors and continuous feeding systems [6,28,29,30]. Through these enrichment strategies, a total of 66 previously uncultured and difficult-to-cultivate microorganisms from diverse environments have been discovered since 2009 (Table 1).

Notable examples include the discoveries of *Candidatus* Manganitrophus noduliforman [23], Chloroflexota [27], *Candidatus* Prometheoarchaeum syntrophicum [29], and TM7x [19]. *Candidatus* Manganitrophus noduliformans is the first bacterium known to grow chemoautotrophically through manganese oxidation. In a study conducted by Yu and colleagues, these manganese-oxidizing bacteria were enriched using a manganese carbonate medium, leading to the identification of this new species [23]. Additionally, Tsuji and colleagues discovered a non-oxygenic photosynthetic bacterium within the Chloroflexota phylum, belonging to an unrecognized order. This discovery was made by employing a previously published freshwater medium and utilizing diuron as an inhibitor of oxygenic phototrophs [27]. Imachi’s team successfully cultivated and studied of *Candidatus* Prometheoarchaeum syntrophicum, marking a significant milestone as the first identification of an asgard archaeon. This study bridged a gap in our comprehension of the evolutionary transition from archaea to eukaryotes. In 2020, this research team used a continuous-flow cell system to enrich and purify deep-sea microbes utilizing methane as an energy source. This innovative approach facilitated the effective isolation and cultivation of *Candidatus* Prometheoarchaeum syntrophicum [29].

The TM7 candidate division, which is associated with periodontal disease, has been one of the most challenging bacterial phyla to study due to the scarcity of cultivated representatives, limiting our understanding of it. However, a novel oral culture medium known as SHI medium, developed by He’s group, supports the growth of many previously uncultivated bacteria in multispecies communities, including several strains of TM7. Using targeted enrichment methods with saliva samples cultured in the SHI medium, stable co-cultures of the TM7 subdivision (TM7x) from the oral cavity were successfully obtained [19]. Li and colleagues devised a novel fungal isolation technique specifically for fungi from mangrove sediments. Their pioneering methods, the fungal enrichment culture method (FECM) and fungal isolation chips (FiChips), enabled the isolation of 660 fungal strains from these sediments. These strains were preliminarily classified into 3 phyla, 9 classes, 23 orders, 41 families, and 64 genera, including 29 potential novel species. The study also described and proposed 38 new ascomycetous taxa, comprising 3 new families, 8 new genera, 25 new species, and 2 new combinations, based on morphological comparisons and phylogenetic analyses [39].

Oligotrophic conditions and extended incubation times have proven to be effective strategies for cultivating previously uncultured microorganisms. Choi and colleagues successfully isolated 20 different taxonomic Gram-negative marine bacteria using dilution-to-extinction culturing. The bacteria, belonging to the phyla Bacteroidetes and Proteobacteria, included some members that may represent a new family [40]. Pulschen’s team developed a method that integrates the use of an oligotrophic medium, long-term culture, stereomicroscopic observation, and the selection of slow-growing bacteria. This innovative method led to the successful isolation of rare microorganisms from Antarctic soil samples, including uncommon or newly described genera such as *Lapilicoccus*, *Favitalea*, *Quadrisphaera*, *Motilibacter*, and *Polymorphobacter* [41].

Furthermore, using an infrared fluorescence imaging system, over 5000 bacterial colonies from various water bodies were screened. *Gemmatimonas* sp. AP64, a unique strain from the freshwater Swan Lake in the western Gobi Desert, contains a functional photosynthetic reaction center but does not fix inorganic carbon [42]. In the same year, Gavrish’s team utilized a variety of cultivation methods such as serial dilution, microscopic observation, and colony enrichment culture to isolate and purify the strain *Lentzea kentuckyensis* sp. IS009804 from soil samples. This led to the discovery of lassomycin, an antibiotic effective against *Mycobacterium tuberculosis* [43]. In 2020, Wiegand et al. leveraged a suite of cultivation techniques, including selective suppression preparations, selective nutrient media design, and microscopic observation, to successfully culture and characterize 79 bacterial species from the phylum Planctomycetes [44].

### 2.2. In Situ Cultivation

For microbial species with unclear nutritional requirements, cultivation can be achieved by using the natural environment as the ultimate culture medium [45]. Scientists have developed innovative techniques for cultivating microorganisms, both in simulated natural environments and in situ (within their natural habitats). Among these cutting-edge methods are the isolation Chip (iChip) [31,46,47] culturing chip (cChip) [48], diffusion growth chambers [7,49,50,51], hollow-fiber membrane chambers (HFMC) [52], and Microencapsulation [53,54]. These advanced techniques are designed to more accurately replicate natural conditions, thereby enhancing the cultivation of microorganisms that were previously difficult or impossible to grow.

Among these techniques, the iChip has demonstrated remarkable efficacy in isolating microorganisms from soil that were previously unculturable. A significant achievement using this method was the discovery of a new class of antibiotics called teixobactin [46]. In 2015, Ling et al. used the iChip culture device to isolate an uncultured strain from the phylum *β*-Proteobacteria, tentatively named *Eleftheria terrae*. This strain led to the identification of teixobactin, a novel antibiotic that effectively kills a broad spectrum of pathogens without detectable resistance [31]. Furthermore, three new antibiotics—amycobactin, streptomycobactin, and kitamycobactin—were isolated from uncultured *Amycolatopsis* sp., *Streptomyces* sp., and *Kitasatospora* sp., respectively [46]. Using the diffusion chamber method, Remenár’s team isolated a total of 260 strains from nickel-contaminated soil in Slovakia, representing 108 species across six bacterial phyla, with 29.6% of these isolates being previously uncultured bacteria [7]. In 2023, Liu and colleagues developed a method combining urease gene enrichment with in situ culture using microspheres. Single cells were encapsulated in agarose microspheres, enriched for target flora with the functional gene *ureC*, cultured in a simulated rumen environment, and identified through genome sequencing. This method successfully isolated urealytic bacteria from the rumen, showcasing the potential of these advanced techniques to open new avenues in microbial research and antibiotic discovery [55].

### 2.3. Metagenomics-Based Approach

Although cultivation-dependent approaches have successfully uncovered many previously uncultured organisms, they typically focus on a narrow subset of easily cultivable species, leaving the vast majority of the microbial world unexplored. As a result, these methods still have significant limitations in fully capturing the breadth of microbial diversity. High-throughput sequencing technology allows scientists to rapidly acquire DNA sequences from a vast number of microorganisms, including those that have not been cultured. The term “metagenome” was coined in 1998 by Jo Handelsman and collaborators to emphasize the importance of soil microorganisms as sources of novel natural compounds [56,57]. Subsequently, Kevin Chen and Lior Pachter defined metagenomics as a method for accessing bacterial genes directly from environmental samples, bypassing the need for traditional culturing techniques [58]. In 2004, Jillian Banfield utilized random shotgun sequencing of DNA from an acidophilic biofilm to reconstruct near-complete genomes of *Leptospirillum* group II and *Ferroplasma* type II [59]. This groundbreaking study is widely regarded as a pivotal moment in the emergence of metagenomics, showcasing its potential to unveil microbial diversity at an unprecedented scale. The field gained significant momentum in 2010 with the development of a comprehensive reference gene set for the human gut microbiome through metagenomic sequencing. This milestone marked the official onset of the metagenomic sequencing era, enabling deeper insights into microbial communities and their functional potential in various environments [60]. With ongoing advancements in next-generation sequencing (NGS) technology and bioinformatics tools, metagenomic sequencing has emerged as a cornerstone of microbial research. This powerful approach allows for comprehensive analysis of environmental samples, revealing the diversity, functional potential, and interactions of microbial communities without the need for prior cultivation [61,62,63,64].

Craig Venter pioneered the sequencing of environmental genomes from microbes in the Sargasso Sea, uncovering over 1.2 million previously unknown microbial genes. This landmark study significantly advanced the understanding of marine microbial communities and their genetic diversity [65]. Employing Stable Isotope Probing (SIP) and amplicon sequencing, Cai et al. identified an uncultured *Gammaproteobacterium* sp. as a pivotal organism in naphthalene degradation [35]. Similarly, Wang et al. discovered the uncultured bacterium *Acidovorax* sp. WH, which plays a dominant role in breaking down naphthalene in contaminated groundwater, highlighting the critical roles of uncultured microbes in environmental bioremediation [66].

An uncultured bacterium associated with low methane emissions was identified from the microbiota of *Macropus eugenii* through metagenomic data analysis. Pope et al. classified this bacterium as a species of the Succinivibrionaceae family, designated WG-1 [67]. Lugli et al. combined specific culture techniques and metagenomics to isolate and purify two novel *Bifidobacterium* species, named 2028B and 2034B, from animal stool samples [68]. In another study, a previously uncultured thermophilic spirochete, *Longinema margulisiae* gen. nov., sp. nov., was successfully isolated from deep subsurface aquifers in Western Siberia, further expanding the catalog of microbial diversity from extreme environments [38].

Using metagenomics techniques, Wilson et al. analyzed the coexisted bacteria in the marine sponge *Theonella swinhoei* and proposed a new candidate phylum named Tectomicrobia [69]. Tianero et al. isolated metagenomic DNA from four Halichlona sponges and conducted deep metagenomic sequencing. Through flow cytometry and cell sorting, they discovered a symbiotic bacterium named *Candidatus* Endohaliclona renieramycinifaciens and identified the biosynthetic gene cluster (BGC) for renieramycin in its genome [70]. In a separate study, two rounds of sequencing were performed on different DNA extracts from Forcepia species sponges collected in the Gulf of Mexico. This led to the discovery of a Gram-negative bacterium named *Candidatus* Thermopylae lasonolidus, belonging to the phylum *Verrucomicrobia* and identified as a candidate producer of Lasonolide A [71]. Additionally, metagenomic sequencing revealed *Candidatus* Didemnitutus mandela, a previously uncultured verrucomicrobial symbiont of the tunicate *Lissoclinum* sp. The genome of this bacterium was found to contain multiple copies of biosynthetic gene clusters responsible for synthesizing secondary metabolites that provide ecological benefits to its host [72].

Paoli’s team investigated the diversity and novelty of BGCs in marine environments by integrating data from approximately 10,000 microbial genomes derived from cultivated strains and single-cell sources with over 25,000 newly reconstructed draft genomes from more than 1000 seawater samples. Their analysis led to the identification of a previously uncharacterized lineage, *Candidatus* Eudoremicrobiaceae, which is notably rich in BGCs and represents up to 6% of ocean microbial communities [73].

In summary, metagenomics can identify and analyze complex biosynthetic gene clusters, predict the structure and function of novel secondary metabolites, and enable targeted screening of gene clusters. Additionally, by constructing and screening libraries using large-fragment vectors such as Cosmid, researchers can efficiently clone and heterologously express candidate gene clusters, thereby obtaining structurally novel antibiotics and other bioactive compounds.

### 2.4. Single-Cell Sequencing

In 2013, single-cell genome sequencing (scGS) was recognized as “Method of the Year” by Nature Methods, underscoring its transformative impact across multiple research fields. This innovative approach has found wide applications in microbial sequencing, haplotype analysis, and cancer research, enabling the detailed study of individual cells and providing insights that were previously inaccessible through traditional bulk sequencing methods [74]. The process of single-cell genome sequencing begins with isolating individual cells, which can be achieved using technologies such as microfluidics [28,75], micromanipulation [76] and fluorescence-activated cell sorting (FACS) [52,77,78,79]. Once cells are isolated, whole genome amplification, library construction, and high-throughput sequencing are performed to generate single-cell genome data [6,80,81,82]. Over the past decade, sequencing the genomes of individual microbial cells directly isolated from environmental samples has become a widely used technique, allowing for deeper insights into microbial diversity and functionality [83].

By combining ^13^C-labeled biphenyl with single-cell analysis and protein-stable isotope probing techniques, the *Alphaproteobacteria* clade UBA11222 was identified as playing an important role in the biodegradation of biphenyl in contaminated soils [33]. Seeleuthner et al. organized the metagenomic data from aquatic samples collected during the Tala Ocean Expedition. Their analysis provided valuable insights into the genome content and distribution of seven prevalent lineages of uncultured heterotrophic stramenopiles [84].

Martinez-Garcia et al. used flow cytometry sorting to isolate individual uncultured protozoan cells from coastal water samples. Their study identified *Pelagibacter ubique* associated with a MAST-4 protist, an actinobacterium linked to a chrysophyte, and three Bacteroidetes associated with various protist groups [85].

Chijiiwa’s group successfully derived 346 microbial single-cell amplified genomes (SAGs) from mouse gut microbiota using the high-throughput single-cell genomic sequencing platform SAG-gel [86]. By using scGS technology, samples from nine different environmental habitats were analyzed and 201 single-cell genomes were obtained. The majority of these genomes belonged to underrepresented categories, including Omnitrophica (OP3) and the phylum Lentisphaerae [87]. In another study, Ngugi et al. documented *Candidatus Nitromaritima* RS, a highly divergent organism from the type species *Nitrospina gracilis* (with a 69% pairwise genome identity), through phylogenetics, scGS, and metagenomic fragment recruitment approaches [88].

To sum up, single-cell sequencing technology has significantly advanced the discovery of microbial natural products, especially novel antibiotics. It can directly obtain complete biosynthetic pathways from rare or uncultivable microorganisms, overcoming the limitations of traditional cultivation methods. Identifying new species using novel techniques like metagenomics and single-cell sequencing technology holds profound significance for evolutionary studies. These techniques enable the discovery of previously uncultured and phylogenetically unique species that fill critical gaps in the tree of life, providing key insights into the evolutionary transitions between major taxonomic groups and revealing ancient evolutionary relationships that were previously obscured by the limitations of traditional cultivation methods [83].

### 2.5. Challenges and Future Directions in Microbial Exploration Methods

**Figure 1:**
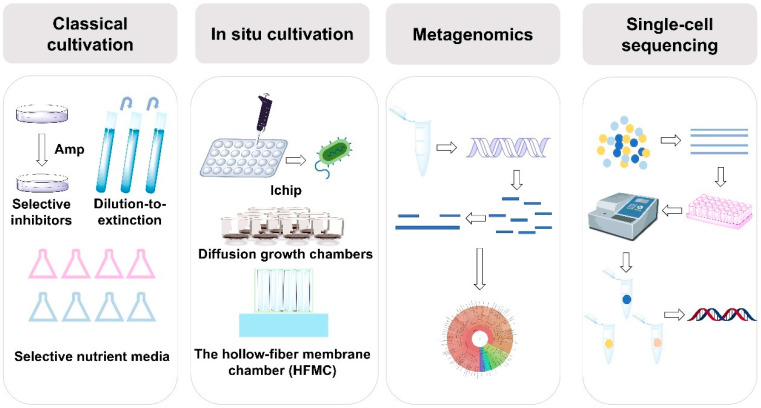
Strategies for cultivating uncultured microorganisms. Classical cultivation methods rely on physiological, phenotypic, and functional characteristics of microorganisms for their isolation. In situ cultivation techniques, such as I-chip, diffusion growth chambers, and hollow-fiber membrane chambers (HFMC), facilitate the direct cultivation of microorganisms in their natural environments. Metagenomics enables the analysis of microbial communities through direct sequencing of environmental DNA, bypassing the need for cultivation. Single-cell sequencing focuses on extracting DNA from individual cells for sequencing to uncover microbial genetic diversity.

**Figure 2:**
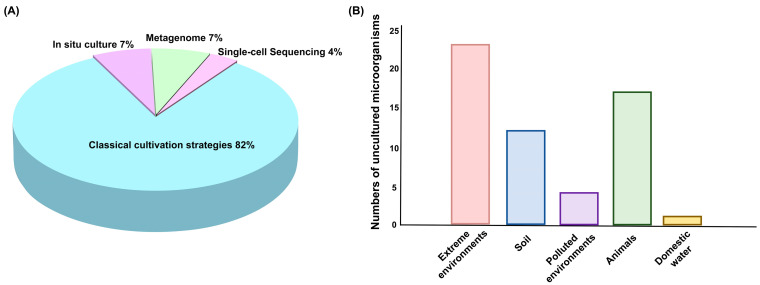
Research strategies and sources of uncultured microorganisms. (**A**) Proportion of strategies used to study uncultured microorganisms. (**B**) Sources of uncultured microorganisms.

Together, these methods—classical cultivation, in situ cultivation, metagenomics, and single-cell sequencing—provide a comprehensive and complementary toolkit for discovering and studying uncultured microorganisms (Figure 1). These approaches enhance our ability to explore microbial diversity, ecology, and biotechnological potential. Over the past decade, classical cultivation strategies have remained the primary method for studying uncultured microorganisms, accounting for 82% of research efforts (Figure 2A). However, modern techniques, such as in situ cultivation, metagenomics, and single-cell sequencing, are increasingly emerging as crucial tools. Among the various sources of the strains, extreme environments—such as deep-sea vents, hot springs, and arid deserts—are home to the highest diversity of uncultured microorganisms (Figure 2B). Extreme environments, such as those with high temperatures, salinity, or pressure, promote the evolution of unique microbial species with specialized metabolic pathways and novel biochemical properties. Animals and soils also host a wide variety of microorganisms that play critical roles in ecological processes and symbiotic relationships. However, polluted environments like contaminated soils or industrial waste sites, and domestic water sources, tend to have fewer species due to selective pressures from pollutants or altered conditions. Despite this, they remain valuable for discovering microorganisms with potential for bioremediation and other specialized applications. However, these methods also have their limitations. Classical cultivation strategies have insufficient ability to simulate microbial interaction dependencies and specific nutritional requirements, leaving a large number of microorganisms that rely on complex community environments unisolable. It also has long cultivation cycles and low efficiency, which cannot meet the needs of high-throughput research. In situ cultivation techniques are constrained by environmental conditions, making the equipment difficult to operate and costly. Metagenomics-based approaches face issues such as DNA extraction from environmental samples being easily interfered by impurities leading to incomplete genome assembly, low sequencing coverage of low-abundance microorganisms making it hard to capture their complete functional information. Single-cell sequencing has low single-cell isolation efficiency, whole-genome amplification is prone to bias resulting in the loss of some gene fragments.

Future efforts to overcome these challenges will focus on multiple technological directions. Innovation through technology integration involves combining microfluidic chips with real-time fluorescence monitoring to develop an “intelligent in situ cultivation system” that dynamically adjusts nutrient supply and physicochemical conditions while tracking microbial growth. It can also involves integrating metagenomics and single-cell sequencing data to accurately capture low-abundance functional microorganisms via “metagenome-guided single-cell sorting”. By engineering model strains simulate the symbiotic environment of uncultured microorganisms to assist in isolating hard-to-culture microorganisms. Tools like CRISPR-Cas9 can be used to edit the BGCs of uncultured microorganisms for efficient expression of natural products in heterologous hosts, thereby solving the problem of “easy gene prediction but difficult product acquisition”. Driven by AI, the nutritional requirements and growth conditions of uncultured microorganisms can be predicted based on existing microbial genomics and metabolomics data to narrow the scope of cultivation condition screening.

## 3. Bioactive Natural Products from Uncultured Microorganisms

Advances in both culture-dependent methods and cultivation-independent strategies for studying uncultured microorganisms hold great promise for unlocking the hidden genetic and metabolic potential of these previously elusive microbes [4,69,89,90]. As researchers increasingly focus on these microorganisms, they are uncovering a treasure trove of novel natural products that exhibit remarkable chemical diversity and a broad range of bioactivities [91,92]. These discoveries are expanding the frontiers of microbial chemistry, revealing new compounds with therapeutic and industrial potential that were once beyond reach due to the limitations of traditional cultivation techniques. Herein, we highlight a selection of non-ribosomal peptides (NRPS), polyketides, and ribosomally synthesized and post-translationally modified peptides (RiPPs), each representing a distinct facet of microbial secondary metabolism (Table 2). These examples not only illustrate the vast untapped potential of uncultured microorganisms that has yet to be fully explored but also demonstrate the broader applications of microbial-derived bioactive molecules in medicine and biotechnology.

### 3.1. Bioactive NRPS from Uncultured Microorganisms

**Figure 3:**
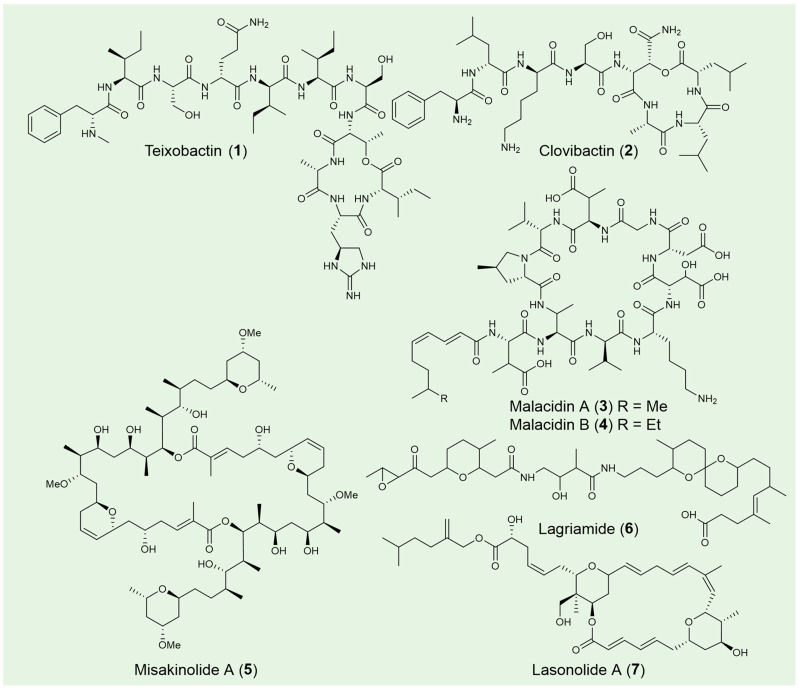
Selected NRPS and polyketide natural products from unculture microorganisms.

Teixobactin (**1**) is a depsipeptide antibiotic discovered from *Eleftheria terrae*, an uncultured Gram-negative *β*-proteobacterium that was identified from a soil sample using the iChip technology [31]. The chemical structure of teixobactin features a distinctive scaffold composed of enduracididine, methylphenylalanine, and four *D*-amino acids (Figure 3). These unique components contribute to the antibiotic’s structural divergence from existing antibiotics and its potent antibacterial activity, enhancing its potential as a promising therapeutic agent, especially in the context of antibiotic-resistant infections. Teixobactin is highly effective against a broad range of pathogenic microorganisms including drug-resistant *Staphylococcus aureus*, *Streptococcus pneumoniae*, *Enterococci*, *M. tuberculosis*, *Clostridium difficile* and *Bacillus anthracis*. Potent in vivo efficacy was also observed in mice models of infection with *S. aureus* and *S. pneumoniae* [98]. Mechanism of action studies suggest that teixobactin separately targets lipid II (peptidoglycan) and lipid III (teichoic acid), which are essential precursors of bacterial cell wall biosynthesis. This binding specificity accounts for teixobactin’s potent efficacy against Gram-positive strains, while also explaining its limited activity against most Gram-negative bacteria [99,100]. The further design and synthesis of teixobactin analogs are currently under development. With continued advancements, these analogs hold the potential to become valuable tools in the fight against antibiotic-resistant pathogens, offering promising new therapeutic options for the future [101].

Clovibactin (**2**) was isolated from the uncultured bacterium *Eleftheria terrae* ssp. carolina in sandy soil following prolonged incubation [93,102]. Its chemical structure features two *D*-amino acids and an uncommon residue, *D*-3-hydroxyasparagine, at its linear *N*-terminus and within the depsipeptide cycle, respectively (Figure 3). Notably, clovibactin has a shorter linear *N*-terminus, consisting of four residues, compared to the seven residues found in teixobactin (**1**). Clovibactin demonstrates potent antibacterial activity against a broad spectrum of Gram-positive pathogens, including methicillin-resistant *S. aureus* (MRSA), daptomycin-resistant and vancomycin-intermediate-resistant *S. aureus* (VISA), as well as vancomycin-resistant *Enterococcus faecalis* and *Enterococcus faecium* (VRE). In contrast to teixobactin, which inhibits cell wall synthesis by binding to lipid II and lipid III, clovibactin targets the pyrophosphate (PPi) of key peptidoglycan precursors, including C55PP, lipid II, and lipid III WTA. It binds tightly to PPi via an unusual hydrophobic interface, bypassing the variable structural elements of these precursors, which contributes to its ability to evade resistance.

Malacidins A (**3**) and B (**4**), two metagenomic acidic lipopeptide antibiotics, were discovered through a culture-independent discovery platform targeting NRPS adenylation domains [94]. These 10-membered cyclic lipopeptides contain peptide cores with four non-proteinogenic amino acids (Figure 3). Notably, malacidins lack the canonical Asp-X-Asp-Gly motif typically associated with calcium binding, and they exhibit no antibacterial activity with other cations, highlighting their unique calcium-dependence.

Malacidin A showed promising antibacterial activity against a range of multidrug-resistant Gram-positive strains, including MRSA and VRE. Malacidin A was also able to clear MRSA skin infection in a rat model, and resistant mutants of MRSA could not be obtained under laboratory conditions. Unlike other calcium-dependent antibiotics, malacidins do not cause membrane depolarization or bind to C55-P. Instead, they interact with lipid II in a calcium-dependent manner. This unique mode of action not only confers potent antibacterial efficacy but also minimizes the risk of resistance development, as evidenced by the absence of resistance even after prolonged exposure. This metagenome-driven discovery platforms highlight the value of mining environmental microbiomes for structurally unique and biologically active compounds, offering promising implications for combating antibiotic resistance.

### 3.2. Bioactive Polyketides from Uncultured Microorganisms

Misakinolide A (**5**), a dimeric macrolide known for its potent cytotoxic properties, is the only polyketide reported from the sponge *Theonella swinhoei* WA [94]. Evidence suggests that this compound is synthesized by the uncultivated symbiont *Candidatus* Entotheonella serta TSWA1 [95]. Architecturally, misakinolide closely follows the *trans*-AT colinearity rules, with the exception of an apparently skipped last module and the yet-to-be-determined formation of the tail-associated tetrahydropyran ring (Figure 3). Misakinolide A binds two actin subunits with similar affinity to swinholide A, another actin inhibitor differing only in macrolide ring size. Both compounds target the actin cytoskeleton, disrupting its organization, and hold potential therapeutic applications in the treatment of cancer and other human diseases. Interestingly, swinholide A severs actin filaments, while misakinolide A merely caps the barbed end of F-actin [103]. This difference may result from a variance in the orientation of one binding site relative to the other, explaining why swinholide A exhibits severing activity while misakinolide A acts solely as a capping agent. Given that capping proteins can either inhibit polymerization or stabilize filamentous actin, misakinolide A could serve as a valuable tool for elucidating capping protein function.

Lagriamide (**6**), an antifungal polyketide produced by the uncultured symbiont *Burkholderia gladioli* Lv-StB, is primarily found in the eggs of the insect *Lagria villosa* [96,104]. The installation of the epoxide, tetrahydropyran, and spiroacetal groups, as predicted, is not carried out by the PKS/NRPS proteins (Figure 3) [96]. Lagriamide inhibits the growth of *Aspergillus niger*, *Beauveria bassiana*, *Metarhizium anisopliae* and exhibits general antifungal activity against *Purpureocillium lilacinum*, a fungus that infects *L. villosa* eggs and serves as a natural enemy to these beetles. Beyond egg defense and generational transmission, the symbiont’s presence during larval development provides protection throughout the molting process [105]. The ectosymbionts, including lagriamide and its producer Lv-StB, inhabit invaginations of the cuticle that form crypt-like structures, offering physical shelter for the symbionts. Positioned externally, these structures ensure that defensive metabolites are readily available to deter antagonists approaching from the environment. Connected to glandular cells, these ectosymbionts remain anchored after molting, unlike typical insect exocrine glands that shed with the exuvia. Long-term vertical transmission and coevolution with the host have led to a substantial reduction in Lv-StB’s genome size, now about one-fourth of that in free-living *B. gladioli* strains, likely allowing for nutrient exchange from surrounding glandular cells to sustain the symbionts [106].

Lasonolide A (LSA) (**7**), originally isolated from the marine sponge *Forcepia* sp. [107], has been shown through genomic analysis to be produced by the uncultivated symbiotic bacterium *Candidatus* Thermopylae lasonolidus, residing within the sponge host [71]. The molecular structure of LSA features two tetrahydropyran rings and *β*-methylations at C-13 and C-35, characteristics commonly associated with *trans*-AT PKS biosynthetic pathways (Figure 3). LSA exhibits subnanomolar anticancer activity, acting through a unique mechanism involving premature chromosome condensation [108], loss of cell adhesion, activation of RAF1 kinase in the Ras pathway [109], and induction of cell blebbing and contraction [110]. Detailed in vivo mechanism discovered that lasonolide A act as a prodrug, becoming cell-permeable through cleavage by lipid droplet-associated hydrolase in the sidechain ester [111]. Once inside the cytoplasm, LSA accumulates and exerts its anticancer effects. This novel mode of action highlights LSA as a promising anticancer drug candidate.

### 3.3. Bioactive RiPPs from Uncultured Microorganisms

**Figure 4:**
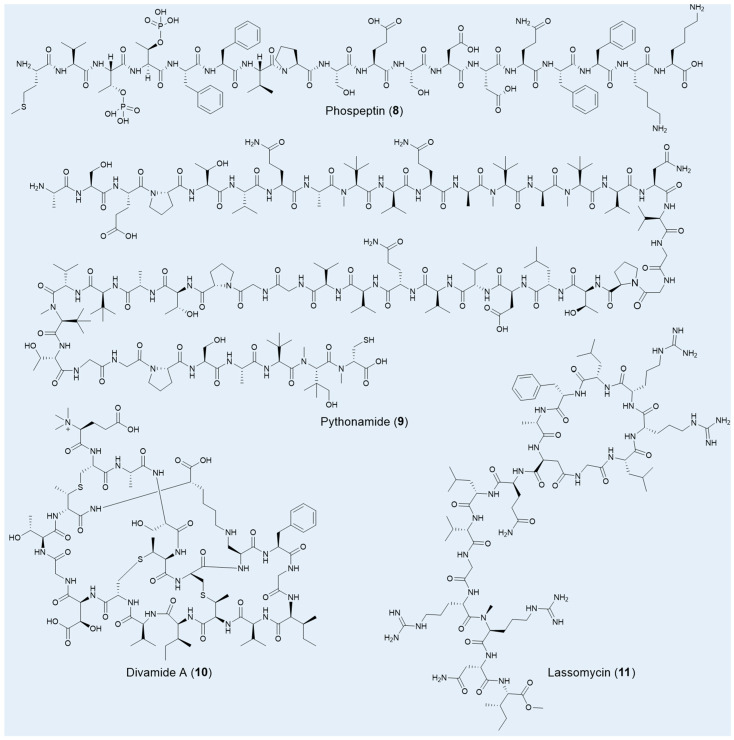
Selected RiPPs natural products from unculture microorganisms.

Using a microbiomics-driven strategy, approximately 40,000 potential new BGCs have been uncovered within vast marine resources. Combined with the heterologous expression method, two RiPPs, phospeptin (**8**) and pythonamide (**9**), were specifically characterized for their novel architectures and significant pharmacological potential (Figure 4) [73]. Both compounds were derived from the uncultured bacterium *Candidatus* Eudoremicrobiaceae, highlighting the immense untapped potential of marine microbiomes for the discovery of bioactive natural products. Phospeptin, a poly-phosphorylated linear peptide catalyzed by a single maturase, demonstrated low-micromolar protease inhibitory activity against neutrophil elastase, with a 50% inhibitory concentration (IC_50_) of 14.3 µM, positioning it on par with other relevant natural products. Pythonamide, on the other hand, stands out for its versatile maturase modifications, including *N*-methylation, *L*- to *D*-amino acid epimerization, and hydroxylation. While N-methylation is common in NRP natural products, its enzymatic occurrence in amide bonds is challenging and biotechnologically significant, with such modifications previously observed only in the borosin RiPP family [112,113].

Another marine uncultured bacterium, *Prochloron* spp., which endows its symbiotic host tunicates with a variety of bioactive, presumably defensive chemicals, remains uncultivated to this day. Leveraging a metagenomic-synthetic biology approach, Thomas E Smith and colleagues unearthed a suite of anti-HIV compounds known as the divamides (**10**), without ever culturing *Prochloron* spp. [97]. These compounds, belonging to the lanthipeptides family, are distinguished by unique structural features, including three methyllanthionines, a lysinoalanine, a *β*-hydroxy aspartic acid, and an *N*-terminal trimethylation—a rare post-translational modification in nature (Figure 4) [113]. Bioassays revealed that divamide A exhibited impressive anti-HIV activity with an IC_50_ of 0.225 μM and demonstrated no cytotoxicity below 10 μM. Further studies revealed that minor modifications in the amino acid sequence could distinguish between cytotoxic and antiviral effects, offering valuable insights for the development of novel antiviral agents.

Lassomycin (**11**), a unique lasso peptide featuring a macrolactam ring and a C-terminal esterification (Figure 4), was uncovered from the previously uncultured soil bacterium *Lentzea kentuckyensis* sp. using a bioactivity-guided screening approach [43]. This peptide demonstrates exceptional bactericidal activity, specifically targeting *M. tuberculosis*, with a MIC of 0.8–3 μg/mL, and exhibits low cytotoxicity. Impressively, lassomycin remains effective against dormant-phase mycobacteria and drug-resistant strains, where even the leading bactericidal agent rifampicin often falls short. Lassomycin’s bactericidal effect is achieved by activating the ATPase ClpC1 without promoting ATP-dependent protein degradation, a mechanism distinct from those previously reported. Interestingly, the total chemical synthesis of lassomycin resulted in a complete loss of bioactivity against *M. tuberculosis*, underscoring the critical role of its natural, unthreaded structure in maintaining its potency [114].

These compounds were primarily discovered through culture-independent metagenomics, enabling access to marine and insect symbionts producing metabolites like misakinolide A (**5**), lagriamide (**6**), lasonolide A (**7**), phospeptin (**8**), and divamide A (**10**). Complementary advanced in situ cultivation and selective suppression methods facilitated the recovery of bioactive agents from soil microbes, notably yielding teixobactin (**1**), clovibactin (**2**), and lassomycin (**11**). Looking forward, single-cell genomics offers a promising avenue to further expand this repertoire by resolving biosynthetic potential at the level of individual, uncultured cells from complex environments.

## 4. Concluding Remarks and Future Perspectives

The exploration of microbial diversity remains one of the most promising frontiers in biotechnology, particularly in the quest for novel natural products with therapeutic and industrial applications. As highlighted in this review, recent breakthroughs in cultivation techniques, metagenomics, single-cell genomics, and synthetic biology have significantly advanced our ability to access the genetic and chemical diversity of previously inaccessible microbial communities. These innovations have facilitated the discovery of new natural products with remarkable metabolic potential, opening the door to novel drugs and biotechnological applications.

AI-driven approaches, particularly in data analysis and pattern recognition, are poised to enhance our ability to predict microbial behavior and uncover new biosynthetic pathways. Machine learning algorithms can rapidly sift through large genomic datasets—such as metagenomic libraries from extreme environments or host-associated microbiomes—to identify promising BGCs with unprecedented efficiency. Additionally, AI can optimize fermentation conditions for natural product production: predictive models can simulate how variables like pH, temperature, and nutrient composition affect microbial metabolism, enabling high-throughput screening of optimal culture parameters to boost yields of target compounds. AI also supports virtual screening of natural product libraries against therapeutic targets, accelerating the identification of lead compounds with desired bioactivities.

Synthetic biology tools allow for the rational design and engineering of microbial strains, enabling the production of valuable compounds at industrial scales. This includes the reprogramming of microbial biosynthetic pathways to improve yield and stability. CRISPR-Cas9 and other gene-editing technologies further enable precise modification of BGCs—such as knocking out competing metabolic pathways or enhancing the expression of rate-limiting enzymes—to increase the production of target natural products. Synthetic biology also facilitates the “design-build-test-learn” cycle for novel compound development: researchers can assemble synthetic BGCs by combining modular components from different microbes, generating hybrid natural products with improved bioactivities.

Currently, the cultivable microbial resources have been extensively exploited and compound rediscovery rates remain exceedingly high. The field of antimicrobial therapy faces a long-term shortage of new antibiotics. Molecular ecology research has confirmed that the vast majority of microorganisms in the environment are “uncultivable”, comprising a treasure trove with phylogenetic and chemical diversity far exceeding our current understanding. Therefore, overcoming the bottleneck of cultivability is no longer merely a technical challenge in microbiology but a critical prerequisite for pharmacology to access novel lead compounds. Successfully isolating and culturing these uncultivable microorganisms aims to unlock their unique biosynthetic gene clusters, thereby providing pharmacology with a steady stream of candidate drug molecules featuring entirely new structures and mechanisms of action. This ultimately offers new hope for addressing the global health crisis of antibiotic resistance.

## Figures and Tables

**Table 1:** Uncultivated and difficult-to-cultivate microorganisms discovered in this review along with cultivation methods and sources.

Representative Taxa	Sources	Classification	Cultivation Methods	Ref.
*Chlorobi*, *Kiritimatiellaeota* and *Marinilabiliales*	Marine	Bacteria	Growth factors	[17]
*Leucobacter ASN212*	Polluted environment	Bacteria	Growth factors	[14]
*Oscillibacter*	Animal	Bacteria	Selective suppression preparations	[16]
*Actinobacterota*, *Deferribacterales*, *Melioribacteraceae*, *Synergistota*, *Ciceribacter Ez67* and *Bradyrhizobium Ez93*	Mineral water deposit	Bacteria	Selective nutrient media	[18]
*Amycolatopsis*, *Lechevalieria* and *Streptomyces*	Desert	Bacteria	Selective agents	[15]
14 potentially novel genera	Animal	Bacteria	Dilution to extinction and selective nutrient media	[21]
*Candidatus* Ethanoperedens thermophilum	Marine	Archaea	Selective physicochemical condition	[25]
The novel lineage of the order Sulfolobales HS-1 and novel species of the genus *Sulfolobus* HS-3	Hot spring	Archaea	Selective physicochemical conditions	[26]
Seven strains of *Alphaproteobacteria*, three strains of *Betaproteobacteria*, one *Gammaproteobacterium* and one *Bacteroidetes* phylotype	Lake, marine and soil	Bacteria	Selective physicochemical conditions	[24]
*Candidatus* Manganitrophus noduliformans	Tap water	Bacteria	Selective nutrient media	[23]
Chloroflexota	Lake water	Bacteria	Selective nutrient media and selective physicochemical conditions	[27]
*Nitrospirota* sublineages I and II	Polluted environment	Bacteria	Bio-devices	[28]
*Candidatus* Prometheoarchaeum syntrophicum strain MK-D1	Marine	Archaea	Bio-devices	[29]
*Candidatus* Promethearchaeum syntrophicum strain MK-D1	Marine	Archaea	Bio-devices	[30]
TM7x	Animal	Bacteria	Selective nutrient media	[19]
*Proteobacteria*, *Acidobacteria*, *Firmicutes*, *Actinobacteria*, *Verrucomicrobia*, *Planctomycetes*, and *Bacteroidetes*	Soil	Bacteria	Selective nutrient media	[22]
*Alphaproteobacteria* SO-S41	Forest	Bacteria	Selective nutrient media	[20]
*Eleftheria terrae*	Soil	Bacteria	In situ culture	[31]
*Amycolatopsis* sp., *Streptomyces* sp. and *Kitasatospora* sp.	Soil	Bacteria	In situ culture	[32]
*Alphaproteobacteria* clade UBA11222	Soil	Bacteria	Single-cell Sequencing	[33]
TM7	Animal	Bacteria	Single-cell Sequencing	[34]
Gammaproteobacterium	Polluted soil metagenome	Bacteria	Metagenomics	[35]
*Chloroflexus* sp. SYSU G00190R	Hot spring metagenome	Bacteria	Metagenomics	[36]
WG-1	Wallaby microbiota metagenome	Bacteria	Metagenomics	[37]
*Longinema margulisiae* gen. nov., sp. nov.	Groundwater metagenome	Bacteria	Metagenomics	[38]

Note: the 14 candidate novel genera identified in this study, along with their closest cultivated relatives and similarity percentages, are as follows: *Ruminococcus albus* (89.3%), *Prevotella dentalis* (88.7%), *Butyrivibrio* (89.1%), strain P6 (90.9–94.5%), *Lachnospira pectinoshiza* (91.9%), *Bacteroides capillosus* (90.4%), *Butyrivibrio hungatei* (90.5–90.6%), *Eubacterium rectale* (89.8–90.9%), *Prevotella ruminicola* (91.4–91.7%), *Butyrivibrio* (92.0%), *Clostridium polysaccharolyticum* (92.2%), Strain YE64 (92.7%), *Bulleidia extructa* (92.4%), *Ruminococcus flavefaciens* (92.6%).

**Table 2:** Selected natural products isolated from uncultured microorganisms in this review.

Compounds	Molecular Formula	Biosynthetic Origin	Producing Microorganism	Sources	Isolation Methods	Ref.
Teixobactin (**1**)	C_58_H_95_N_15_O_15_	NRPS	*Eleftheria terrae*	Soil	In situ culture	[31]
Clovibactin (**2**)	C_43_H_70_N_10_O_11_	NRPS	*E. terrae* ssp. *carolina*	Soil	In situ cultivation and biosynthetic gene cluster disruption	[93]
Malacidin A (**3**)	C_56_H_88_N_12_O_20_	NRPS	Not provided	Soil	Culture-independent strategy	[94]
Malacidin B (**4**)	C_57_H_90_N_12_O_20_	NRPS	Not provided	Soil	Culture-independent strategy	[94]
Misakinolide A (**5**)	C_74_H_128_O_20_	*trans*-AT PKS	*Candidatus* Entotheonella serta TSWA1	Marine	Metagenome	[95]
Lagriamide (**6**)	C_41_H_69_N_2_O_10_	*trans*-AT PKS/NRPS	*Burkholderia gladioli* Lv-StB	Insect	Metagenome	[96]
Lasonolide A (**7**)	C_42_H_62_O_9_	*trans*-AT PKS	*Candidatus* Thermopylae lasonolidus	Marine	Metagenome	[71]
Phospeptin (**8**)	C_100_H_147_N_21_O_36_P_2_S	RiPPs	*Candidatus* Eudoremicrobiaceae	Marine	Metagenome	[73]
Pythonamide (**9**)	C_201_H_342_N_50_O_62_S	RiPPs	*Candidatus* Eudoremicrobiaceae	Marine	Metagenome	[73]
Divamide A (**10**)	C_86_H_136_N_21_O_28_S_3_^+^	RiPPs	*Prochloron didemni* GUM007	Marine	Metagenome	[97]
Lassomycin (**11**)	C_83_H_142_N_30_O_20_	RiPPs	*Lentzea kentuckyensis* sp. IS009804	Soil	In situ cultivation and selective suppression preparations	[43]

## Data Availability

Not applicable.
